# *In vivo *fiber tractography of the right and left ventricles using diffusion tensor MRI of the entire human heart

**DOI:** 10.1186/1532-429X-16-S1-P17

**Published:** 2014-01-16

**Authors:** Choukri Mekkaoui, Timothy G Reese, Marcel P Jackowski, Himanshu Bhat, William J Kostis, David E Sosnovik

**Affiliations:** 1 Radiology, Harvard Medical School - Massachussets General Hospital - Martinos Center for Biomedical Imaging, Charlestown, Massachusetts, USA; 2 Department of Computer Science, University of São Paulo, São Paulo, São Paulo, Brazil; 3 Siemens Medical Solutions USA Inc., Charlestown, Massachusetts, USA

## Background

Diffusion Tensor MRI (DTI) tractography of the human heart *in vivo *has previously been performed with either large slice gaps or limited coverage [1,2]. The aim of this study was to investigate the feasibility of performing DTI of the entire human heart *in vivo *without slice gaps. This, we hypothesized, would enhance the characterization of fiber architecture in the left ventricle (LV), allow myofiber organization in the right ventricle (RV) to be characterized *in vivo*, and further elucidate microstructural differences in the heart between systole and diastole.

## Methods

DTI was performed on a clinical 3T scanner (Skyra, Siemens) using a fat-suppressed, zone-selected, diffusion-encoded stimulated echo sequence with 10 diffusion encoding directions, TE/TR 33/80 ms, GRAPPA rate 2, b-value 500 s/mm^2^, resolution 2.8 × 2.8 × 8 mm^3^, 8 averages and multiple breath-holds. The entire LV and RV were covered in 13 contiguous short-axis slices. Images were acquired in the systolic and diastolic sweet spots [3] of the cardiac cycle and were spatiotemporally coregistered [4]. Tractography was performed by numerically integrating the primary eigenvector field into streamlines using an adaptive 5^th ^order Runge-Kutta method. The impact of cumulative image averages (1-8) on the reliability of the fractional anisotropy (FA) and fiber helix angle (HA) indices was assessed.

## Results

**Figure 1:**
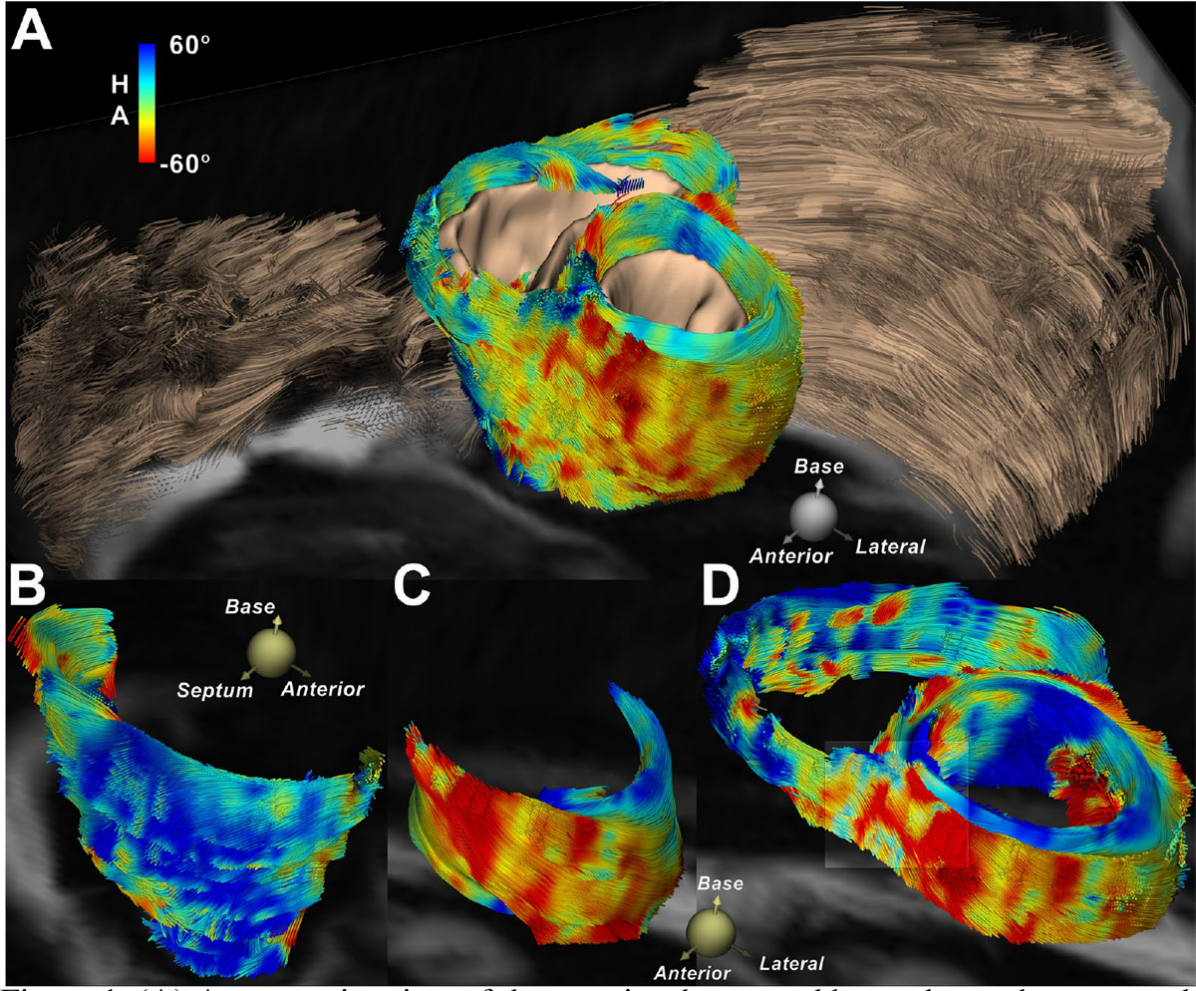
**(A) A composite view of the anterior thorax and heart shows the contrast between the helical pattern of the fibers in the myocardium (color-coded by HA) and the linear organization of skeletal muscle fibers in the chest wall**. (B) The fiber architecture of the RV can be well resolved *in vivo *and lacks the circumferential fibers seen in the LV (C). (D) Tractography within a slab located at the basal-level depicting the transmural myofiber arrangement of both RV and LV. The intertwined arrangement of myofibers at the anteroseptal RV-LV junction is slightly magnified.

**Figure 2:**
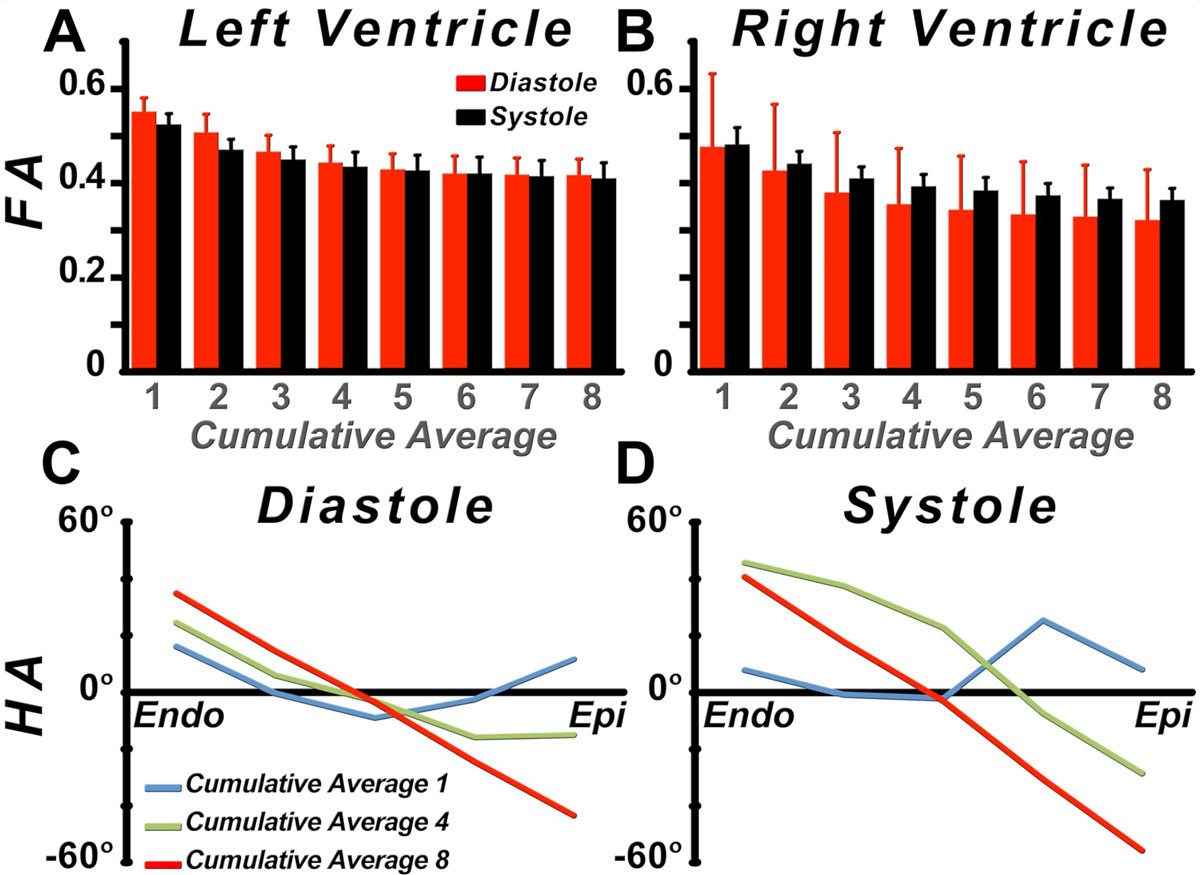
**(A, B) Cumulative averages (1-8) of FA for the LV and the RV, respectively**. (A) In the LV, FA values stabilize after 5 averages in diastole as well as in systole. **(B)** In the RV, FA also stabilizes after 5 averages, albeit with a higher standard deviation in diastole, but FA values are 9% lower in diastole than in systole. (C, D) Transmural distribution of HA for 1, 4, and 8 cumulative averages in diastole and systole, respectively. For optimal estimation of HA, 8 averages are required since the HA is a function of the primary eigenvector, and more degrees of freedom need to be estimated.

A composite view of the anterior thorax and heart is shown in Figure 1A. The contrast between the helical pattern of the fibers in the heart (color-coded by HA) and the linear organization of skeletal muscle fibers in the chest wall can be seen. The RV consisted of a bilayer of obliquely-oriented fibers, lacking circumferential fibers **(**Figure 1B**)**. In contrast, fibers in the midwall of the LV were circumferential, forming a distinct band between the subendocardial and subepicardial fibers **(**Figure 1C**)**. Figure 1D shows the tractogram of the entire heart, depicting the intertwined arrangement of myofibers at the anteroseptal RV-LV junction. Figure 2A and 2B show that FA values in both sweet spots were significantly overestimated when the number of averages used was < 5 (p < 0.01). In contrast, convergence to a stable FA value (LV, diastole: 0.42 ± 0.03, systole: 0.41 ± 0.03; RV, diastole: 0.32 ± 0.11, systole: 0.36 ± 0.02) was observed from 5-8 averages. The transmural gradient in HA exhibited a similar pattern of convergence but required more averages **(**Figure 2C, D**)**.

## Conclusions

DTI-tractography of the entire human heart can be performed *in vivo*, without slice gaps, and allows both the LV and the RV to be characterized. A minimum of 5 averages at each slice location is required for accurate quantification. The ability to characterize fiber architecture in the LV and RV *in vivo *has the potential to provide new insights into a range of diseases affecting both the pulmonary venous and arterial circulations.

## Funding

R01 HL093038 (D.E.S.), R01HL112831 (D.E.S.), P41RR14075 (Martinos Center).
